# Coping with the Challenges of COVID-19 Using the Sociotype Framework: A Rehearsal for the Next Pandemic

**DOI:** 10.5041/RMMJ.10425

**Published:** 2021-01-19

**Authors:** Wen Peng, Elliot M. Berry

**Affiliations:** 1 Department of Public Health, Medical College, Qinghai University, Xining, China; 2 Braun School of Public Health, Hebrew University-Hadassah Medical School, Jerusalem Israel

**Keywords:** Coping, COVID-19, emergency preparedness, government responsibility, health systems, humor, media, public health, sociotype

## Abstract

The world, as a global village, is currently taking part in a real-time public health, medical, socio-cultural, and economic experiment on how best to combat the COVID-19 pandemic. Extraordinary times demand extraordinary measures. Depending on the time from the outbreak, strategies have ranged from minimal intervention to mitigation by quarantine for high-risk groups (elderly with chronic illnesses) to containment and lockdown. Adherence to such restrictions have depended on the individual and national psyche and culture. One can understand and forgive governments for being over-cautious, but not for being ill-prepared. It seems that Singapore after SARS (2003) and South Korea after MERS (2015) learnt from their experiences and have fared relatively well with minimal disruption to daily routines. Coping with the challenge of COVID-19 is an urgent global task. We use the Sociotype ecological framework to analyze different coping responses at three levels: Context (government and leadership, social context, health services, and media); Relationships; and the Individual. We describe the many negative outcomes (e.g. mortality [obviously], unemployment, economic damage, food insecurity, threat to democracy, claustrophobia) and the positive ones (e.g. new, remote teaching, working, and medical routines; social bonding and solidarity; redefining existential values and priorities) of this surreal situation, which is still evolving. We highlight the importance of humor in stress reduction. Regular and reliable communication to the public has to be improved, acknowledging incomplete data, and learning to deal with fake news, misinformation, and conspiracy theories. Excess mortality is the preferred statistic to follow and compare outcomes. When the health risks are over, the economic recovery responses will vary according to the financial state of countries. If world order is to be reshaped, then a massive economic aid plan should be launched by the rich countries—akin to the Marshall plan after the Second World War. It should be led preferably by the USA and China. The results of the tradeoffs between health and economic lockdowns will only become apparent in the months to come. The experiences and lessons learned from this emergency should be used as a rehearsal for the next epi-/pandemic, which will surely take place in the foreseeable future.

## INTRODUCTION

Prediction is very difficult, especially if it’s about the future.

(Niels Bohr)^1^

Land apart, sky shared.

(The Prince Nagoya of Japan)^2^

Since the outbreak of COVID-19 in Wuhan, China, in December 2019, the newly identified corona virus, SARS-COV-2, spread massively worldwide. On January 30, 2020, the World Health Organization (WHO) declared the COVID-19 outbreak a public health emergency of international concern. On March 11, 2020, the WHO announced the pandemic of COVID-19. Most countries and areas have identified cases; an increasing number of people are infected, and measures to tackle the virus are aggressive, including lockdown of countries, deployment of armies, personal surveillance, and more.

The pandemic has caused enormous damage in terms of health, economy, education, and food security. The WHO reported the number of confirmed COVID-19 cases at almost 31 million, with confirmed deaths of almost 1 million as of Septemer 21, 2020.^3^ The International Monetary Fund projected a 4.9% decline in global gross domestic product (GDP) in June 2020,^4^ yet not much recovery has been seen globally. Educationally, nearly 1.2 billion pupils or 68% of the total students enrolled are affected by school closures.^5^ Further, the UN World Food Programme projected that an additional 130 million people could face acute food insecurity by the end of 2020, largely due to disruptions in food trade flow, and this is in addition to the 135 million people who were already food-insecure before the COVID-19 crisis.^6^ The Human Development Index value is also projected to experience the first decrease since the concept was introduced in 1990.^5^

The scenario is surreal and best fitted to a science fiction story that came true. Compared to the responses to Spanish flu in 1918, this is the first global experience dealing with such a rapidly spreading pandemic. According to current data, the mortality rate of COVID-19 (1%–3%) is higher than that of flu (0.1%); however, the disease is less fatal than SARS (10%) and MERS (34%), although apparently more contagious.^7^,^8^ Up-to-date epidemiological data regarding age, sex, and disease status are important for determining the transmission pattern, and to identify and protect high-risk populations—elderly people with comorbidities.^7^

Fear of the unknown leads to existential angst. The difference from other major catastrophes—tsunamis, earthquakes, nuclear plant disasters—is that the whole world has become involved with a new and hitherto unknown threat that is not yet under control. Everyone feels vulnerable, and although the truth is different, we learn from other countries’ experiences in real time. The decline in cases in China suggests that the peak will be reached in 3–4 months. At-risk patients are those who are most susceptible to winter viruses—the elderly with chronic diseases—and this may explain the high mortality in Italy where the average age of the case fatalities is over 79 years. The Italian experience, where the health system practically collapsed, was the alarm signal for many countries to impose lockdown.

This emergency is testing the level of international cooperation and exchange of information, as it is in everyone’s interest to contain and overcome the virus. Measures are being taken daily by governments, scientific society, health systems, and the public, leading to coping strategies such as isolation, mass lockdown, and movement restriction. Though humankind has encountered other corona viruses—the common cold, but also the more fatal SARS and MERS—the fear of the unknown has overwhelmed previous experiences. Due to the great uncertainty in the actual number of infected individuals, because of the many asymptomatic and undiagnosed cases, the initial overestimates of infected cases and mortality rates by scaremongers only magnified these anxieties. These reactions and loss of control were further driven by the competition to upgrade government management strategies, and inappropriate personal behaviors such as panic-buying and hoarding supplies. While governments may be forgiven for being over-cautious in the face of the pandemic, they cannot be excused for being ill-prepared. It seems that Singapore, after SARS (2003), and South Korea, after MERS (2015), learnt from their experiences and have fared relatively well with minimal disruption to daily routines. While coping with the challenge of COVID-19 is an urgent global task, the experiences and lessons from this emergency should be a rehearsal for the next pandemic, which will surely take place. There is already a warning for this.^9^

## COPING RESPONSES ACCORDING TO THE SOCIOTYPE DOMAINS

**Figure 1:**
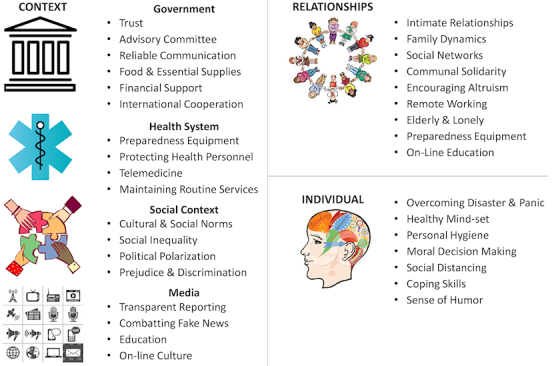
Coping Strategies against COVID-19. Incorporating relevant topics from the social and behavioral sciences, coping strategies can be constructed based on the three domains of the Sociotype framework: Context, Relationships, and Individual. The coping responses in the Context domain are further divided into four sectors: government, health system, social context, and media.

The concept of the Sociotype^10^–^12^ has been developed as an ecological framework to categorize coping responses and strategies. The Sociotype was formulated as an extension of the ecological model of Bronfenbrenner^13^ and the bio-psychosocial framework of Engel.^14^,^15^ It comprises three domains—Individual (intra-personal), Relationships (inter-personal), and Context.^10^,^11^ Individual factors include physical and mental health, personality, and life philosophy. Relationships cover all interactions within the family, friends, and at work. The Context factors include demographics, culture, education, employment, socio-economic position, and national political and health systems.^16^ The Sociotype interacts with the Genotype (heredity) to determine the individual Phenotype which establishes a person’s behavior and cognitive responses to deal with any stress. While the Genotypic background is relatively constant, apart from epigenetic influences, the Sociotypic input is dynamic depending on life experiences. We have previously described how the Sociotype can facilitate coping with food insecurity^16^; we now use its framework to analyze and formulate coping strategies for COVID-19 (summarized in Figure 1) to analyze their outcomes (summarized in Table 1) and to propose relevant contingency plans for the future.

**Table 1:** Outcome Responses to the COVID-19 Pandemic by the Sociotype Domains.

Domains	Side Effects: Negative Fallouts	Side Effects: Positive Fallouts
Context	Threats to democracy—issues of freedom of movement and privacy Closure of institutions—law courts, government in the interests of “public health” Damage to tourism, hotel, restaurant, and service industries Collapse of performing arts, concerts, theatre, etc. Collapse of Stock Market—shades of 1929 recession DALYs lost due to unemployment and stresses in the long term Civil unrest	Benefits to the environment, decreased pollution, especially from less air traffic Decrease in terrorism—less crowds Exercise in preparedness, especially if a really serious disease such as a variant of Ebola emerged Research into the immunity of bats as a reservoir for zoonotic illnesses New organizational solutions, e.g. transformation of hotel rooms as quarantine stations; restaurants to food kitchens Advancing implementation science to find vaccines and new, quicker diagnostic tests Closer international cooperation and sharing data and experiences; use of Big Data analytics Developing new online experiences—museum tours etc.
Relationships	Loss of normal work routines Loss of employment—loss of self-respect, financial consequences Caring for the food-insecure—closure of food banks Disruption in work and recreation activities Lovers not being able to meet… Effect on divorce rate?	Experiencing family quality time—getting to know family/kids better Getting back to, and appreciating basics—friendships, nature Social empathy Learning how to use technology and to build virtual communities for mutual support, encouragement, and boosting morale Revised educational methods and teaching platforms, leading to internet teaching and reduction in frontal lectures (long overdue) in schools and universities Re-organization of working schedules to allow for remote working from home; encouraging use of virtual meetings—this will directly and indirectly affect transportation and traffic congestion Advances in the use of telemedicine Effect on birth rate—baby boom?
Individual	Unavoidable loss of life—especially among the elderly with chronic diseases Time seems to stand still, a feeling of being in limbo Ageism Inability to exercise—weight gain, obesity Living without cultural events and live sports Increased stress and anxiety Inability to be on one’s own, or being confined with someone with whom you do not get on Screen time addiction	Learn about time management Time to do all those things we never have any time to do Stress management Existential issues and change in priorities Smoking cessation?

In order to prepare materials for this paper, we used the key words “COVID-19,” “SARS-COV-2,” “coping,” “response,” “pandemic,” “epidemic,” and their combinations to search PubMed and Google Scholar for relevant information in the scientific literature. In addition, we followed updates about COVID-19 in reputable non-academic journals, e.g. *Financial Times*, and the official webpages of international organizations, such as World Health Organization, Food and Agriculture Organization, World Bank, and the International Monetary Fund, etc., and of governments, such as Singapore. Also, information was gleaned from real-life experiences and personal communications. Useful references and messages from these resources were traced back further with often interesting novel insights.

### What Can We Learn from Previous and the Current Pan(Epi)demics?

It is of interest to compare the Sociotype approach to dealing with COVID-19 to that of medical sociology. In 2013, a whole issue of *Sociology of Health and Illness* (volume 35, number 2) was devoted presciently to “Why a Sociology of Pandemics?” Eleven articles were analyzed with special reference to coping and resilience. There was only one reference to coping,^17^ and that was in the context of the cholera and syphilis epidemics in the nineteenth century. The concept of resilience was mentioned in two papers.^18^,^19^ In both, the term referred to institutions (the Context domain of the Sociotype). Steyer and Gilbert discussed risk management policies focusing on external shocks, a country’s resilience, and business continuity management for vital infrastructures.^19^ Surprisingly, the article by Godderis and Rossiter on volunteers, specifically during the 1918 influenza pandemic, did not consider either coping or resilience.^20^ The articles in this issue give a comprehensive historical perspective, including responses to avian flu and West Nile Virus. Of special note is the detailed contribution by Sherlaw and Raude on the actions by the French authorities and the public to the A/H1N1 pandemic in 2009.^21^ The discussion highlights innovations diffusion theory, surprise theory, and social representation theory. Unfortunately, it is not possible in this paper to consider the interrelationships between these theories and coping and resilience strategies at all levels of the Sociotype in society. The challenge remains to integrate both perspectives. The Sociotype assesses the many aspects and players involved in coping strategies of the individual and society. Figure 1 and Table 1 are an attempt to itemize the very many *practical* considerations of the different responses to COVID-19 and their possible outcomes according to the three Sociotype dimensions. This is all the more difficult since the situation and the scientific literature are constantly evolving.

In previous infectious disease outbreaks (SARS, Ebola, and H1N1), common psychological responses were anxiety/fears, depression, anger, guilt, grief and loss, post-traumatic stress, and stigmatization, but also, more positively, a greater sense of empowerment and compassion toward others.^22^,^23^

Coping approaches have included problem-focused coping (seeking alternatives, self- and other-preservation), seeking social support, avoidance, and positive appraisal of the situation.^22^ Other techniques involved behavioral activation, acceptance-based coping, mindfulness practice, and loving-kindness practices (altruism).^23^ Initial reports from China suggested that unmarried people with negative coping styles displayed higher levels of psychological distress in the early stages of COVID-19 epidemic.^24^

Practical advice has included focusing on the individual in the larger social environment, to explain the range of possible psychosocial responses, providing psychological help, self-care, empowering self-support groups, and regular access to updated, reliable information about the outbreak.^22^ Such effective and rapid communication is essential for people who are quarantined to understand the situation. Supplies need to be provided with special attention to ensuring food security. Most of the adverse effects of quarantine come from restriction of liberty, and voluntary quarantine was associated with less distress and fewer long-term complications. Public health officials should emphasize the altruistic choice of self-isolation.^25^

Taken together, these approaches help individuals make meaning, build distress tolerance, increase social support, emphasize deep human interconnectedness, and take goal-directed value-driven actions. What is not clear is how to develop these skills in real time at the population level.

We also mention here the problem of the responses of health workers and caregivers to dealing with overwork and burn-out.^26^ To alleviate their stress, Schwartz rounds (eponymously named after a patient who noted that “the smallest act of kindness made the unbearable bearable”) have been recommended. These discussions are introduced to allow the entire hospital personnel—doctors, nurses but also porters, catering staff, pharmacists, librarians, managers, and administrators—to freely express feelings and concerns about the care of particular patients.^27^

Finally, a most comprehensive perspective on the responses to COVID-19 has now been published by 38 authors from the social and behavioral sciences.^28^ The summarized responses can help align human behavior with the recommendations of epidemiologists and public health experts as well as lessen individual psychological suffering. Six principal themes are discussed: threat perception; leadership; individual and collective interests; science communication; social context; and stress and coping.^28^ Each has a number of sub-topics (20 in all) which may be incorporated easily within the Sociotype framework, as shown in Figure 1.

We realize that the world community is part of an on-going learning experience in dealing with the current pandemic. There is no formula or operating manual for coping with the current global pandemic. It has disrupted nearly every aspect of daily living, with quarantining, social distancing, fears of lethal infection, and feelings of helplessness and hopelessness, as well as economic hardship which increases with the duration of the shutdowns. We will all be far wiser and able to evaluate and prioritize the best “solutions” at each level when the pandemic has burnt itself out. None of the literature cited above deals with the current situation in a holistic manner to bring in *all* levels of involvement—national, inter-personal, and intra-personal—which is the task and role of the Sociotype. The lessons from these and other publications will now be considered in the framework of these Sociotype domains.

### Responses in the CONTEXT Domain in the COVID-19 Pandemic

The coping responses in the Individual, Relationship, and Context domains are dynamic and have to adjust in real time to the current situation. The early reactions were principally in the Context domain in preparing for, and preventing, viral spread. At the phase of local transmission, coping measures became more general with extensive interactions between the three domains. Figure 1 lists these coping responses, which are taken mainly from the countries with moderate to high-performance health systems. Though experience from and for resource-constrained countries and areas was accumulating, including mobilization and engagement of the wider society, early establishment of a formal responding committee, etc.,^29^,^30^ relevant information is still limited. Capacity constraints, such as accessibility to health care and testing facilities,^31^ have probably masked the actual situation when the virus is spreading naturally.

The preparedness of the health system and governmental leadership are critical for adequate and appropriate coping responses, and responsible reporting from the media is crucial. What is encouraging is the on-going fast, international knowledge sharing among the scientific community,^32^ from virology and epidemiology to clinic management, particularly in this otherwise polarized world.

#### Responses by the government and leadership

The role of government leadership and the confidence and trust by the public are essential for coordinating resources and raising public morale in such an emergency.^28^

The unprecedented decision to lock down Wuhan city and other cities in Hubei Province (population 60 million) and the measures in other provinces have been proved retrospectively to be effective. Here, public trust is very important when such seemingly over-zealous measures are instituted. In these situations, the preparedness of the health system and the media’s role in accurate and responsible reporting are essential. Introducing aggressive measures, if implemented immediately by a seemingly over-cautious government, may not be readily accepted or followed by the public. Group psychology using “successive approximation” for behavioral change helped the population to accept decrees and limitations that they would otherwise refuse. This involves introducing restrictions gradually such as cutting down the numbers at a gathering from say 5000 to 1000 to 100 to 10 to lockdown. What is not clear is how sustainable such measures are, and for how long they may be maintained. This will depend on the specific country, its social cohesion and culture, coping skills and resilience.

By contrast, the Singapore government tried to maintain minimal interruption to normal life and has a fairly satisfactory control of COVID-19. Preparedness might be the reason. Singapore has a color-coded classification framework, called “Disease Outbreak Response System Condition” (DORSCON), to show the level of risks of infectious diseases and corresponding measures to be taken in daily life.^33^ The current status (as of August 7, 2020) is equivalent to the SARS epidemic in 2003.^34^ Meanwhile, measures to prevent imported cases are enforced strictly—for example, airport fever screening from the beginning of January, compulsory reporting of, and compulsory quarantine after, international travel, and other travel restrictions.^35^ More than 800 public health preparedness clinics,^36^ which were established by equipping ordinary community clinics after the SARS epidemic in 2003, serve as the gatekeeper against outbreak of infectious diseases. Social responsibility is also addressed by the government.^36^ While countries in Asia and Europe such as India, Nepal, Spain, and the UK are almost competing for implementing drastic measures, these seemingly simple measures in Singapore showed the critical importance of preparedness in both public health infrastructure and timely interventions for successful disease control. This has flagged Singapore as a positive deviant country in coping.^37^

The responses by some other countries and areas, such as Japan, Hong Kong, Taiwan, and South Korea, are also to be commended.^38^ Of interest is that the current zoonotic pandemic was predicted by Afelt et al.^39^ However, organizational silos may impede coping with these “hybrid” diseases.^40^ Therefore, some international organizations, such as the World Health Organization, the Food and Agricultural Organization, and the World Organization for Animal Health, adopted the “One World, One Health” policy framework in the A/H1N1 outbreak to advance organizational actors in various areas, and to maximize their own legitimacy and influence.^41^ Such a framework will hopefully promote the sustainable development between human and other species. The principles in this framework seek to define a holistic approach to the prevention of epidemic/epizootic diseases, while maintaining the integrity of ecosystems for the benefit of mankind, our domestic animals and biodiversity, a topic that concerns us all. Such a framework will hopefully promote the sustainable development between human and other species.

Because of the rapidly changing situations, coping policy adjustments are needed based on the best available knowledge and experiences. The upgrading of prevention strategies in Europe (e.g. mobile cabin hospital building, lockdown of cities and even countries, travel and movement restriction, remote working, school suspension, etc.) are examples of such policies. South Korea, which was one of the earliest epicenters in the outbreak, showed a sharp decline in newly confirmed cases following such measures. Large-scale diagnostic testing was implemented, and more than a quarter of a million people had been tested by March 15, 2020.^38^ Other actions, such as compulsory screening of specific groups who were blamed for accelerating virus spread in the early stages as well as application of transformed medical support and observation centers for mild cases of disease, also contributed to controlling the pandemic.^42^ Current concern is now focusing on dealing with “the second wave.”

There is also an issue of the structure of the *senior health advisory team*, how this team communicates with the public, whether the government accepts their recommendations, and what other competing interests (such as economics, unemployment, routine medical care for patients with chronic illnesses) the government needs to prioritize. The team requires a multi-disciplinary background in public health, communication techniques, population psychology, clinical medicine, and basic sciences. They should work according to risk management protocols.^41^,^43^,^44^

Instructions should be provided that are easily understood, reasonable, and may be carried out by the public over a long period (months). Much more work is needed to improve communication messages.

Psychological health often depends on financial security. Governmental roles in fiscal and monetary policies are therefore highly important in compensating for the economic decline, massive unemployment, and recession following shutdown—topics which are, however, beyond the scope of this paper. Interested readers are referred to two recent reports published by Schmidhuber and colleagues at the FAO on the effects on food and agriculture and the economic fallouts.^45^,^46^

#### Responses in social context

The current pandemic has highlighted that morbidity and mortality are disproportionately higher in the lower socio-economic groups and ethnic minorities.^47^,^48^ Inequalities are also driving the social unrest which has long been simmering and recently exploded in the Black Lives Matter protests as a response to structural racism which is now considered to be a public health emergency of global concern.^49^ While it is difficult to prove a direct causal relationship with the current pandemic (although this has been postulated by Galea and Abdalla),^50^ social inequalities and the differential ethnic morbidities have added urgency to such rallies which have spread to many countries. It is to be expected that these demonstrations and others fueled by increased economic hardships will only escalate unless urgent social support programs become effective.

#### Responses by the health system

The health system acts professionally in terms of strategy, public instruction, public health, and clinical management. The selection of action strategies—the containment, mitigation, or natural spreading—is based on the dynamic balance between the burden on the health system and the resources available to cope with it, before a vaccine or suitable drug treatment is developed.

Containment is a powerful initial intervention involving epidemiological tracing of all patients and their contacts and cutting off all the transmission chain identified. Efforts are made to ensure that every patient is isolated, traced, tested, and treated. From the lockdown of Wuhan on January 23, 2020, China applied the containment strategy nationwide, with the coordinated national resources from health and many other sectors. Massive efforts were made particularly in Hubei. Closed community management was employed, and whole-population screening by symptoms was conducted in Wuhan. To admit and provide medical care to all the patients, two designated hospitals were built, 16 mobile cabin hospitals transformed from hotels, and exhibition halls and dormitories provided more than 20,000 temporary beds for patients with mild disease; some 42,000 health workers (doctors and nurses) were directed to Hubei to assist. Insurance and government covered all medical costs. These strategies, together with the collective power and self-discipline of the population, helped control the outbreak.

By contrast, case mitigation is a compromised option due the insufficient resources available to stop the spread or delay in reaction to the epidemic. It includes the quarantine of high-risk, vulnerable people (usually the elderly with chronic illnesses), with the goal of flattening and extending the epidemiological curve, in order to avoid overloading the health infrastructure, hospital beds, respirators, and intensive care facilities. The UK and some European countries initially applied mitigation until modeling predicted an unacceptable steep rise in cases and fatalities. Meanwhile, the implosion in Italy was a “wake-up call”^51^ against mitigation in favor of containment policies.

Some countries with weak public health infrastructures and a very low reported number of cases, such as in parts of Africa, adopted a naturally spreading pattern. Yet, due to the limited resources, some effective measures cannot be deployed in these settings where testing capacity and medical support are inadequate. The instructions to the public, such as hand washing, social distancing, and compulsory or voluntary quarantine, are difficult to implement because of resource constraints, such as water availability and personal protective equipment, and crowded living conditions. Disadvantaged populations probably are, and will be, the most badly affected by this pandemic. Therefore, finding drug treatment and speeding up vaccine development are the most urgent priorities to protect the vulnerable.

There is also an issue of integrating public health and clinical management approaches. It is necessary to separate the management of mild cases from hospitalized patients with serious disease, to prevent overload of the health system and minimize nosocomial infections. Data from China and Italy have shown tragic infections in health personnel (more than 3300 cases in China and 20% of responding health workers in Italy), which should have been avoidable.^52^ The public health preparedness clinics in Singapore function as the first line in screening to prevent overload of hospitals and nosocomial infections.^36^ Drive-through testing in South Korea decreased close contact, and this approach has been adopted by many other countries.^42^

In addition, regular health care has to continue for the many with chronic non-communicable diseases (NCDs), and also for handling emergencies other than COVID-19 cases. Online medical consultation including psychological support should be provided, as done in China. Continuous international collaboration, e.g. multi-center clinical trials, vaccine development, and knowledge and experience sharing, are also important. Such collaborative efforts to combat the common threat are precious in a politically divided world.

#### Responses by the media and TV

Social responsibility and professionalism of the media in the emergency are critical for public communication and trust. However, with the constant obsessional number counts (led by Reuters), the population feels very threatened. This senseless and gruesome body counting and country rankings are only for the morbidly curious, as though there were some kind of sport competition between countries, bringing to mind the Maya games where the “winners” were allegedly executed and deified. Annually, some one billion people catch “regular” flu and between 290,000 and 650,000 die. If numbers were that important, why is there indifference to the plight of refugees in Syria? About 6.7 million Syrians are now refugees, and another 6.2 million people are displaced within the country, half of whom are children. The media choose selectively and poorly. This underlines the importance of responsible reporting and on-going health literacy; reporting has been, until now, more sensational than carefully prepared. Transparent but not scary reporting on the number counts and case fatalities is necessary.

Communication to the public has to be improved, acknowledging incomplete data, especially about the ambiguous denominator due to the asymptomatic or undiagnosed cases, and avoiding the use of “percentages of percentages.”^53^ The recommended form of reporting has been reviewed recently. Compared with the other data available, excess deaths are the best indicator of the mortality impacts of the pandemic. Excess deaths also reflect the state of the outbreak several weeks previously given the long course of infection.^54^ In Europe, the response by Sweden will be used as the “control” scenario where no lockdown measures were taken. As of June 1, 2020, the excess mortality in Sweden was not as low as the other Scandinavian countries (Denmark 6%, Norway zero), but was similar to the USA, France, and Switzerland (approximately 26%) but less than Spain (56%), the UK (49%), and the Netherlands (31%).^8^ Other data should be presented not by total cases but preferably per million inhabitants for meaningful inter-country comparisons.^7^,^8^ Also, only when the economic consequences are factored into the outcomes will the true picture and optimal strategies emerge.

Meanwhile, the measures undertaken and progress made should also be reliably reported to ease public panic and psychological stress. The government, as well as the senior health advisory team, should take the responsibility for directing and dominating the media discussion and providing trusted and sensible information. Staniland and Smith^55^ have discussed the media’s role in the infectious disease outbreak from a sociology standpoint. From the practical perspective, more details on, and reasons for, implementation need to be discussed openly. Full media coverage should be made available to the whole population including the lonely and vulnerable, who may have limited access to TV and reading material. Traditional channels, with radio broadcasting, may be operated as in China for rural villages.

Fake news and conspiracy theories encourage sensationalism and panic. Authoritative media platforms to identify misinformation are essential, as were developed in China by social media, collaborating with government and professional teams. The Singapore government had an official ministry of health webpage to clarify the misinformation on COVID-19.^34^ Besides fake news, unclear modeling with alarming results is another source of generating panic. In fact, most people do not realize that the modeling results are dependent on the assumptions given and the data available, which cannot accurately reflect all the factors in reality. Though model-based evidence was called on during the A/H1N1 pandemic outbreak in 2009,^56^ both models and evidence—the two competing philosophies of scientific knowledge—need to be integrated.^57^ The collapse in the 1990s of Long-Term Capital Management, who were advised by Nobel Economics Prize winners with the highest skills in financial modeling and risk management, showed the limitations of predicting the future.

In addition to fake news identification, the social media in China also bridged the public needs and government policy modifications, particularly in the lockdown situation in Hubei. Real-time adjustments in NCD management, group purchasing of life supplies, screening strategies, enrollment procedures, etc. were made as the information accrued. Finally, the point to make is that the media must be a partner to the responsible communication of messages to the public that take into account cultural sensitivity and health literacy. The latter is most relevant as shown by the unacceptable increase in non-adherence to vaccination instructions; this could be a public health problem when a COVID vaccine is operational.

### Responses in the RELATIONSHIPS (Inter-personal) Domain in the COVID-19 Pandemic

Family cohesion and shared experiences provide mutual support to cope, both physically and psychologically, with the challenges of the pandemic. Child management with limited access to recreational facilities and at home 24/7 may be relieved by remote group activities within schools, with friends, and using YouTube films on different activities. Interactions among friends and colleagues through social networking—WhatsApp, Facebook, Skype, Zoom, Wechat, Weibo, etc.—provide a whole new way of interacting, both socially and professionally.

Communal solidarity at different levels helps allaying feelings of loneliness. During these difficult times, Chinese opera students who trained in Italy sang Puccini to the Italians^58^; Italians in voluntary quarantine sang “Va, pensiero”—the chorus of the Hebrew slaves from Nabucco (Verdi, 1842)—on their balconies.^59^ Initiatives coming from social empathy and responsibility emerged everywhere. Volunteers actively serve the community and public through household visits and screening, supply and delivery chain maintenance, locating and contacting lonely people living on their own, and assisting in transportation for health workers.

Educationally and culturally, many institutions are now producing new online products and experiences—from sport, opera, concert, and theatre performances, museums, schools, universities, and more. However, there is tremendous concern for the future employment of artists, actors, and musicians—in effect, the whole of the cultural scene.

Professional services, such as medical consultations including psychological support, are moving online where feasible. Electronic commerce is developing further. Instead of restaurant dining, food delivery services are having a greater share of the market.

### Responses in the INDIVIDUAL (Intra-personal) Domain in the COVID-19 Pandemic

The very first reaction in the Individual domain might be the shift toward a war/siege mentality. Hoarding and stockpiling behaviors, e.g. food, medicine, masks, toilet papers, etc., may assist in easing the fear of insecurity, yet are unacceptable and even shameful.^28^ After the first psychological shock, social responsibility is perhaps the best guide for choosing personal behaviors. Trust in the authorities and their instructions, personal hygiene, and social distancing need to be followed. Self-discipline in voluntary quarantine is protective to individuals and to vulnerable populations and encourages altruism. These socially responsible behaviors are, in turn, positive inputs to the Relationship and Context domains.

There are also challenges for individuals in coping with isolation, claustrophobia, and living with uncertainty and fear. Coping resilience is related to personality, which sometimes is classified in the five-personality traits model of “OCEAN.”^60^ The collective personality of a country or nation mirrors its culture and social norms in the Context domain. These should be important factors in response strategy developments at policy levels. Thus, adherence to restrictions depends on the individual and national psyche and culture.

Reframing is another positive thinking framework in the Individual domain. The extended home stay creates time to do the jobs on the long waiting list. This is also the chance for more family activities. Sense of humor helps to alleviate the stress.^61^ The often black humor spontaneously created by the public has brought smiles to many faces (see Box 1).

Box 1 **Examples of Cross-Cultural Corona Jokes and Humorous Memes as Relief**

❖ Drinking Corona beer
❖ “You know what goes great with the Corona virus? Lyme disease”
❖ “If there is no toilet paper, let them use tissue—Marie Antoinette”
❖ Video: Man walks inside a two-meter hula hoop for social distancing
❖ God gives Adam hand gel from the ceiling of the Sistine chapel
❖ Wearing decorative and fun face masks
❖ Video: Woman dances gleefully on hearing that her husband is in quarantine
❖ “Corona Virus sitting in Pub: My vision is not to KILL people per se, but to raise awareness around access to public health. Customer: $&@# millennials …”
❖ “Covid is just a bad case of flu’ with great PR”
❖ “All those people panic buying, make sure you stock up on condoms, so you don’t produce any more idiots”
❖ Song taking off the Beatles—“I gotta wash my hands”
❖ Reframing Social DisDANCING and Social DistanSING
❖ And many, many more

## OUTCOMES OF RESPONSES TO THE COVID-19 PANDEMIC

The coping responses to control the COVID-19 spreading, such as isolation, social distancing, and remote working, etc., have their fallouts as trade-offs to the direct beneficial health consequences. Some are negative, such as the inevitable impacts on the economy and recession which will affect the coping capacity in all three domains of the Sociotype. With a more optimistic mind-set, there may also be some positive consequences. Some temporary measures undertaken now point toward new ways of doing things in the future, such as telemedicine, online education, and a new awareness of society and of the fragility of the world as with climate change. Table 1 lists the outcomes of the coping responses to COVID-19, both negative and positive, based on the Sociotype domains.

Economic recession is an inevitable negative outcome in the Context domain. Nevertheless, the calculation of loss is not only limited to the economy. Because of the increasing unemployment and consequent increased stress, the DALYs (disability-adjusted life years) lost are also relevant, but the data may take years to appear. Drastic fiscal and monetary policies have already been implemented for economic stimulus by major countries such as the USA and China. Paradoxically, this might be a chance to develop and scale up novel online business models, for example, online consultation services, education, museum tours, performing arts, and more. However, there is also concern over the current tools and technologies used for disease surveillance, and that some of the aggressive measures for disease control may pose threats to democracy and personal privacy.^62^

Positive contextual fallouts also exist. Less road and air traffic, with decreased atmospheric pollution, has obvious benefits to the environment. Terrorism probably also has decreased because of less crowds and competing national and regional priorities. There is tremendous stimulus to implementation science for improved diagnosis, treatment, and vaccine development. Closer international cooperation has led to sharing of data and experiences. Prestigious scientific journals and presses have granted free and rapid publication for research on COVID-19, but sometimes at the cost of allowing political considerations to interfere with scientific rigor and due diligence (as in the evolving *Surgisphere* saga with retraction of articles published in *The Lancet* and *New England Journal of Medicine*).^63^,^64^ Inter- and intra-personally, positive and negative outcomes also exist, as listed in Table 1. Of note is that vulnerable populations will increasingly suffer until the COVID-19 pandemic is under control. The loss of employment directly threatens the livelihood of households. The closure of food banks and of schools that provided free meals to children will increase the level of food insecurity.^16^ People who work in temporary jobs and are paid daily will lose their income to support their families in a lockdown situation. The World Bank has projected that COVID-19 could push 71 million people into extreme poverty in 2020 under the baseline scenario and 100 million under a downside scenario.^65^ The vulnerable population suffers most in terms of both health and the negative fallouts from the coping responses, which will contribute to increased frustration, and may lead to economic and civil unrest.

## CONTINGENCY PLANS FOR THE FUTURE

It is hoped that the outcome of the current pandemic will not be totally adverse. In fact, there may be a new realignment of values and priorities. Many of the positive and negative outcomes listed in Table 1 are still on-going. These considerations are essential for future preparedness. To this end, there are several issues that should be related to; the following points are relevant to such planning, though the time line will vary from country to country.

### The Short Term

With regard to short term issues, the following actions are needed:

- Provide medical aid to the countries and areas with weak public health infrastructure to end the battle globally.
- Draw up contingency plans to promote economic recovery at the national and international level.
- Set up social and food support systems for marginalized populations—homeless, elderly, sick, and lonely.

### The Long Term

With regard to long term issues, the following actions are needed:

- Appoint a statutory Advisory Committee to deal with emergency disaster planning and monitoring and risk management; draw up detailed response plans for the next possible pandemic, such as for example Singapore’s DORSCON plan.
- Strengthen health infrastructure in terms of disease monitoring and surveillance, clinical management capacity, research, human capital reserve, and emergency supplies and equipment.
- Strengthen global network for alerts, information sharing, responsible communication, and collaboration.
- Draw up guidelines needed for developing countries without regular access to water for hand washing.
- Activate collective power from the civil society, e.g. local community and volunteer organizations; monitor social media for fake news and misinformation.

## CONCLUSIONS

Looking ahead to when the COVID-19 pandemic is over, we should already be making plans for “the morning after.” Estimates vary wildly as to when that may be, from months to years.

“Business as usual” should not be an option, as the world enters a new phase of alertness and international cooperation and dependency. The fight against COVID-19 has shown for the first time that the world is indeed a global village with reciprocal responsibilities to deal with this pandemic. Since “we are all in the same storm,” the pandemic has raised a tremendous feeling of solidarity and international cooperation which is hoped to lead to a new and braver world.

In thinking about a recovery program, it is clear that rich countries (e.g. USA, Germany, and China) will return to normal relatively quickly. For others less fortunate, the process will be long and slow—even taking a number of years. If there is to be a new world alliance, then it is surely not too naïve to suggest that a massive economic aid plan be launched by the rich countries—akin to the Marshall plan adopted by the USA after the Second World War to help Western Europe. This time many more countries should be involved, and it should be led preferably by the USA and China.

We are currently in the midst of a live experiment and learning experience in sociology and public health epidemiology, preparedness, and responses, the outcome of which is unknown. Meanwhile there is the uplifting feeling that (to paraphrase Voltaire’s *Candide* and follow Leibnizian optimism) “Everyone is doing their best in the best of possible ways.” Yet, when the pandemic is finally over, one hopes that the world will be more prepared for the next one that will surely come. Therefore, the answer to the question whether this pandemic will be a transformational moment in world history depends on whether we press the restart button or install a new upgraded operating system—the latter is to be preferred.

Whatever will be the result of all these major re-adjustments to life and society, humankind will feel like Coleridge’s Ancient Mariner:

He went like one that hath been stunned,

And is of sense forlorn:

A sadder and a wiser man,

He rose the morrow morn.

(Coleridge)^66^

## Abbreviations

DORSCON: disease outbreak response system condition
NCD: non-communicable disease
WHO: World Health Organization
